# Protease-Activated Receptors and other G-Protein-Coupled Receptors: the Melanoma Connection

**DOI:** 10.3389/fgene.2016.00112

**Published:** 2016-06-15

**Authors:** Rebecca A. Rosero, Gabriel J. Villares, Menashe Bar-Eli

**Affiliations:** ^1^Biology Department, University of St. Thomas, HoustonTX, USA; ^2^Department of Cancer Biology, University of Texas MD Anderson Cancer Center, HoustonTX, USA

**Keywords:** GPCR, melanoma, chemokine, protease-activated receptors, cancer

## Abstract

The vast array of G-protein-coupled receptors (GPCRs) play crucial roles in both physiological and pathological processes, including vision, coagulation, inflammation, autophagy, and cell proliferation. GPCRs also affect processes that augment cell proliferation and metastases in many cancers including melanoma. Melanoma is the deadliest form of skin cancer, yet limited therapeutic modalities are available to patients with metastatic melanoma. Studies have found that both chemokine receptors and protease-activated receptors, both of which are GPCRs, are central to the metastatic melanoma phenotype and may serve as potential targets in novel therapies against melanoma and other cancers.

## G-Protein-Coupled Receptors

**FIGURE 1:**
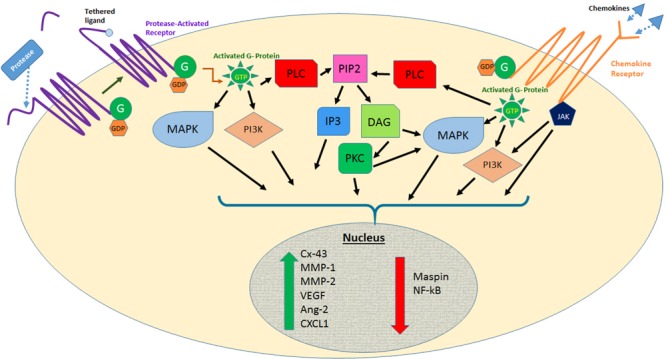
**Effects of protease activated receptor and chemokine receptor activation on several signaling pathways involved in melanoma metastasis.** Abbreviations: MAPK, mitogen-activated protein kinase; PI3K, phosphoinositide 3 kinase; JAK, Janus kinase; PLC, phospholipase C; PIP2, phosphatidylinositol-bisphosphate; IP3, inositol triphosphate; DAG, diacylglycerol; PKC, protein kinase C; Cx-43, connexin-43; MMP, matrix metalloproteinase; VEGF, vascular endothelial growth factor; Ang-2, angiopoietin-2; CXCL1, CXC chemokine motif ligand 1.

The discovery of the 7-pass transmembrane G-protein-coupled receptors (GPCRs) has proven vastly beneficial in the development of modern medicines as nearly half of all drugs today act on GPCRs (Tautermann, 2014). More than 850 GPCRs are found in the human body and function as key mediators in signal transduction pathways from the cell exterior (Tautermann, 2014; Liu et al., 2016). Through specific interactions between the GPCR and the heterotrimeric G-protein molecular switches (G-alpha, G-beta, G-gamma), numerous intracellular signaling pathways are modulated, affecting various physiological processes including vision (Palczewski et al., 2000), blood clotting (Huang et al., 2007), cellular proliferation (Yu and Brown, 2015), and autophagy (Wauson et al., 2014) among others (**Figure 1**).

GPCRs also have specific roles in human disease, which make them excellent targets in drug development. For example, neural pathways that would produce symptoms of schizophrenia are disrupted by the antipsychotic mediators Clozapine and Risperidone, which block specific GPCRs that normally bind to serotonin (Fribourg et al., 2011). Deregulation of Beta-adrenergic receptors, a type of GPCR, is commonly found in various cardiomyopathies (Kamal et al., 2012). Moreover, GPCRs have also been named as key regulators of tumor progression and metastasis through various intracellular pathways deregulated in cancers, including MAPK and PI3K/AKT pathways (Dorsam and Gutkind, 2007).

The wide range of functions affected by GPCRs, including GPCR’s ability to bind a variety of ligands, such as peptides, antibodies, and small molecules, has resulted in the extensive utilization of agonists, antagonists, and inverse agonists (Richard et al., 2001; Feigin, 2013).

## GPCRs and Cancer

Young et al. (1986) discovered and characterized a novel oncogene that encoded a GPCR, which they named *mas*. These were the first experiments demonstrating the GPCR’s transformative activity in tumorigenesis. Since then, many GPCRs have been identified as being involved not only in tumorigenesis but also in metastasis and angiogenesis. In fact, many tumors rely heavily on GPCRs for these other processes, including the protease-activated receptor (PAR), prostaglandin receptors, sphingosine receptors, and the CXCR2 and CXCR4 chemokine receptors (Richard et al., 2001).

Mutations affecting GPCRs can cause abnormal increases in receptor expression and activity that can ultimately lead to the development of cancer. The range of mutations from GPCRs can affect basal activity, ligand binding, GPCR-G protein interaction, cell surface expression, GPCR signaling, and disease (Stoy and Gurevich, 2015). Recently, GPCR mutations, copy number alterations, differential gene expression, and methylation changes have been discovered by genomic analyses in a variety of cancers (Feigin, 2013). This information could potentially lead to the utilization of GPCR-targeted therapeutics in patients with GPCR-driven tumors.

Moreover, various cell types stimulate cell proliferation by potent mitogens such as thrombin, lysophosphatidic acid, gastrin-releasing peptide, endothelin, and prostaglandins, acting on their cognate GPCR. Studies have even shown that wild-type GPCRs have the capability to become tumorigenic upon exposure to an excess of locally produced or circulating ligands and agonist (Julius et al., 1989; Gutkind et al., 1991). Additionally, mutations in key conserved residues can render the GPCRs constitutively active independent of the presence of their ligands (Allen et al., 1991; Pollock et al., 2003).

Although GPCRs are involved in many cancer types, herein, we will discuss the involvement of several GPCRs known to contribute to tumor growth and metastasis of human melanoma. Although melanoma is the fifth most common cancer type in men and seventh in women, it is the deadliest form of skin cancer (Siegel et al., 2016). Even with the discovery of several immunotherapeutic drugs that have had positive effects on patient survival rates, the number of drugs available for advanced metastatic disease remains limited. Thus, GPCRs seem to offer another potential therapeutic modality for the treatment of melanoma (Valko-Rokytovska et al., 2016). Moreover, there is evidence that GPCRs are involved in resistance to RAF and MEK inhibitors in melanoma harboring a common mutation in BRAF (V600E; Johannessen et al., 2013). As such, targeting GPCRs may be important not only for preventing melanoma progression but also in treating drug-resistant melanoma.

### GPCRs and Melanoma

GPCRs are involved in tumorigenesis and metastatic progression of melanoma in all stages, including transformation, proliferation, migration, invasion, and angiogenesis (Li et al., 2005; Lee et al., 2008). In several human cancers, including non-small cell lung cancer, breast cancer, prostate cancer, gastric cancer, and melanoma, the most implicated GPCRs are endothelin receptors, chemokine receptors, and lysophosphatidic acid receptors. Not only are GPCRs up-regulated in primary tumors but they have also been found in metastatic cancer cells. Further analysis of cancer samples in different disease stages showed that some GPCRs, such as endothelin receptor A, may be involved in early tumor progression while other GPCRs, such as CXCR4, are critical for tumor invasion and metastasis (Li et al., 2005). Melanoma studies have revealed a myriad of GPCR and mutations found in GPCRs that may be involved in melanoma progression including, melanocortin type 1 receptor, endothelin receptor, metabotropic glutamate receptor-1, 3, and 8, platelet-activating factor receptor, and Wnt/frizzled receptor among others (Lee et al., 2008; Prickett et al., 2011). In this review, we will concentrate on two families of GPCR that have been identified as being crucial for the progression and metastasis of melanoma: chemokine receptor family and PAR family members.

### Chemokine Receptors

Chemokines are small polypeptide signaling molecules that bind to and activate the G-protein-coupled chemokine receptor. They have the unique ability to induce chemotaxis of leukocytes, but can produce a wide range of cellular signals that affect cell proliferation and promotion of angiogenesis (Payne and Cornelius, 2002).

Chemokine receptors have been found to be involved in cancer progression in various cancers, including melanoma. Various studies looking at signaling pathways in metastatic melanoma suggest that the expression of chemokine receptors and chemokines may grant melanoma cells the ability to inhibit an immune response and alter the tumor microenvironment to stimulate angiogenesis (Lee et al., 2008). Studies on melanoma cell adhesion, survival, and chemo attraction have further elucidated chemokines’ particular role in directing site-specific metastasis. Different metastatic sites express a gradient of varying chemokines. As such, melanoma cells expressing a particular chemokine receptor will preferentially metastasize to sites based upon receptor detection of the chemokine gradient elaborated by a specific organ or tissue microenvironment (Pinto et al., 2014; Valko-Rokytovska et al., 2016). For example, CCR7, CCR10, and CXCR3 have been associated with a greater risk of lymph node metastasis while CXCR4 has been associated with pulmonary metastasis (Pinto et al., 2014). Studies have also shown that CXCL8, CXCR1, and CXCR2 have been associated with tumor progression in melanoma by means of affecting the growth of the tumor cells, angiogenesis, and metastasis (Bar-Eli, 1999).

### Protease-Activated Receptors

Unlike typical ligand-binding interactions that occur with other types of GPCRs, PAR activation occurs through proteolytic cleavage of the N-terminal domain of the receptor by serine proteases. This cleavage causes an irreversible conformational change in the receptor creating a new amino terminus that acts as a tethered ligand to activate the receptor (Vu et al., 1991). Four members of the PAR family have been identified (PAR-1, -2, -3, and -4). These PARs differ in the specific proteases that activate them and their localization.

#### PAR-1

The first PAR to be identified was the thrombin receptor PAR-1 (Vu et al., 1991). It is expressed on a wide variety of cell types, such as platelets, endothelial cells, fibroblasts, neurons, astrocytes, T-cells and smooth muscle cells, among others (Macfarlane et al., 2001). Furthermore, PAR-1 has been found to be expressed by a wide range of cancer cells, including, but not limited to, breast, colon, prostate, and melanoma. Upon activation, the PAR family stimulates multiple intracellular signaling pathways including the MAPK and PI3K pathways that affect crucial cellular processes including cell proliferation and survival, thereby making PAR activation one of the essential components of functioning cells. As such, PAR-1 has significant roles not only in coagulation, wound healing and inflammation, but also in the progression of several cancer types (Jin et al., 2016).

In melanoma, the development of tumor cells is directly dependent on the interaction between the cells and the inhabited microenvironment. A key player in this communication is the thrombin receptor (PAR-1; Tellez et al., 2003). Thrombin activation precedes a coagulation deluge that leads to cell proliferation, cell adhesion, angiogenesis, and invasion, which ultimately promotes tumor growth and metastasis (Villares et al., 2008; Zigler et al., 2011). *In vivo* targeting of human PAR-1 with the use of siRNA-encapsulated liposomes showed a decrease in melanoma growth and experimental metastasis, as well as a decrease in the expression of several angiogenic and metastatic genes, including vascular endothelial growth factor (VEGF), interleukin 8 (IL-8), and matrix-metalloprotease 2 (MMP-2; Villares et al., 2008). Similar findings have not only been seen in *in vivo* and *in vitro* studies, but also in patient samples using tissue microarrays (TMA). Quantitative analysis of a melanocytic microarray showed a significant increase in PAR-1 in metastatic melanoma samples and primary melanoma as compared to dysplastic nevi (Tellez et al., 2007).

Further studies in our lab elucidating the role of PAR-1 in melanoma growth and metastasis found that PAR-1 affects Maspin and Connexin-43, two other genes involved in cancer cell growth and metastasis. Maspin is a tumor-suppressor gene found in melanoma and other cancers such as breast cancers. It was found that increased PAR-1 expression and activity found in metastatic melanoma cells, negatively regulates Maspin expression and promotes the metastatic melanoma phenotype as evidenced *in vivo* when Maspin was overexpressed in PAR-1 silenced cells (Villares et al., 2011). Concomitantly, PAR-1 was found to increase the tumor promoting-function of Connexin-43, a gap junction intracellular communication (GJIC) protein that was found to increase melanoma cell attachment to endothelial cells, thereby augmenting the metastatic phenotype in melanoma (Villares et al., 2009).

These studies on melanoma, as well as other studies on pancreatic cancer and PAR-1 (Queiroz et al., 2014), demonstrate a potential therapeutic benefit in targeting PAR-1. Interestingly, 25 years after the first PAR discovery, an antagonist targeting this receptor was approved in 2014 for human use (Vorapaxar) for the prevention of thrombotic cardiovascular events (French and Hamilton, 2016). Whether this drug can help patients with melanoma metastasis is yet to be determined.

#### PAR-2

Although there is a high degree of homology to PAR-1, PAR-2 is not activated by thrombin, but mostly by trypsin and other trypsin-like proteases, such as tryptase and factor Xa (O’Brien et al., 2001; Bahou, 2003). The tethered ligand domain of cleaved PAR-1 has been found to transactivate the PAR-2 receptor when both are present in close physical proximity to one another (O’Brien et al., 2000). Thus, cross-activation of PAR-2 by PAR-1 could facilitate PAR-1s effect on melanoma metastasis.

The effect of PAR-2 on tumor progression is thought to be due to an increase in chemotaxis mediated by the high levels of calcium released by PAR-2-activated cells (Yau et al., 2016). Moreover, activated PAR-2 results in increased inflammatory conditions via activation of the NFkB pathway with concomitant decrease of anti-inflammatory, tumor-suppressor microRNAs, including let-7d, miR-23b, and miR-200c (Johnson et al., 2016). As inflammation is a risk factor for tumorigenesis, activation of PAR-2 can regulate proliferation, angiogenesis, and metastasis (Johnson et al., 2016).

#### PAR-3 and PAR-4

Both PAR-1 and PAR-3 are thrombin receptors. Studies have shown that PAR-3 mediates the effect of thrombin to produce the cell-adhesion molecule VCAM-1 and cytokine production in endothelial cells as well as cell proliferation of malignant B cells (Kalashnyk et al., 2013). However, studies have found that activated PAR-3 does not result in signaling through G-proteins when PAR-4 is not present (Nakanishi-Matsui et al., 2000). PAR-4 directly plays a role in platelet activation by thrombin as it is required for platelet pro-coagulant functions during thrombus formation (French and Hamilton, 2016). The pro-coagulant environment of circulating tumor cells supports metastases. As such, therapeutics targeting some of the molecules of blood coagulation activation, such as coagulation proteases and PARs, could positively affect patient survival (Lima and Monteiro, 2013).

## GPCRs as Therapeutic Targets in Cancer

The ubiquitous nature of GPCRs in regulating multiple essential cell functions and diseases has resulted in more than 50% of drugs today targeting GPCRs including chemokine receptors and PARs. For example, the use of the chemokine receptor antagonist Maraviroc is an effective HIV antiviral drug that blocks the chemokine CCR5 which is essential for HIV entry into host cells (Dorr et al., 2005; Fatkenheuer et al., 2005). Vorapaxar and Atopaxar are PAR-1 antagonists used in patients with thromboembolism that reduce the risk of cardiovascular death and myocardial infarcts (Capodanno et al., 2012).

Despite the promising results from many GPCR-targeting drugs in various diseases, the development of cancer therapeutics targeting GPCRs has only started to become more mainstream within the last 5 years. There are currently several clinical trials that are testing GPCR-targeting drugs for different types of cancer. Vantictumab, a monoclonal antibody that targets the GPCR Frizzled, an essential protein in Wnt signaling, is in several phase I clinical trials: Vantictumab plus docetaxel for recurrent non-small-cell lung cancer; Vantictumab plus Paclitaxel and Gemcitabine for stage IV pancreatic cancer and Vantictumab plus Paclitaxel for recurrent and metastatic breast cancer. Other clinical trials include the use of the non-steroidal anti-inflammatory drug (NSAID) Sulindac in combination with anti-VEGF antibody (Bevacizumab) and anti-EGFR inhibitor (Erlotinib) for the treatment of squamous cell carcinoma of the head and neck. A new clinical trial is recruiting patients with colon cancer to determine the expression of cannabinoid receptor, a GPCR, as compared to patients with inflammatory bowel conditions. Most of these studies are ongoing or have just been completed and the results from these trials will shed light on the effectiveness of targeting these GPCRs as possible cancer therapies.

## Conclusion

Although tumor progression and metastasis is a multi-factorial process, one of the key players in these cancer processes is the GPCR. In melanoma and other cancers, GPCRs, including chemokine receptors and protease-activated receptors, are crucial in favoring a more pro-metastatic phenotype. Chemokines and their respective GPCR receptors promote angiogenesis and organ-directed metastases, while PAR activation induces a more inflammatory, pro-coagulant environment and controls key cell signaling pathways involved in melanoma growth and metastasis. Given the experimental data obtained in recent years on the effects of GPCRs in cancer, the potential in developing therapeutics to combat them has increased exponentially and could lead to better patient outcomes.

## Author Contributions

All authors listed, have made substantial, direct and intellectual contribution to the work, and approved it for publication.

## Conflict of Interest Statement

The authors declare that the research was conducted in the absence of any commercial or financial relationships that could be construed as a potential conflict of interest.
